# Survival benefits of salvage surgery for primary lung cancer based on routine clinical practice

**DOI:** 10.1111/1759-7714.13961

**Published:** 2021-05-04

**Authors:** Katsutoshi Adachi, Hiroaki Kuroda, Masayuki Tanahashi, Motoshi Takao, Yasuhisa Ohde, Kohei Yokoi, Tomohito Tarukawa

**Affiliations:** ^1^ Department of Thoracic Surgery Mie Chuo Medical Center Tsu Japan; ^2^ The Chubu Lung Cancer Surgery Study Group Chubu Japan; ^3^ Department of Thoracic Surgery Aichi Cancer Center Hospital Nagoya Japan; ^4^ Division of Thoracic Surgery, Respiratory Disease Center Seirei Mikatahara General Hospital Hamamatsu Japan; ^5^ Department of Thoracic and Cardiovascular Surgery Mie University School of Medicine Tsu Japan; ^6^ Department of Thoracic Surgery Shizuoka Cancer Center Sunto‐gun Japan; ^7^ Department of Thoracic Surgery Nagoya University School of Medicine Nagoya Japan

**Keywords:** lung cancer, multicenter study, salvage therapy

## Abstract

**Background:**

Premeditated induction chemotherapy followed by surgical resection is accepted as safe and effective. Studies on salvage surgery in patients with incompletely cured lung cancer are lacking. This study aimed to demonstrate the safety and efficacy of salvage surgery.

**Methods:**

We conducted a retrospective multi‐institutional cohort study on patients who underwent salvage surgery for advanced (stage III and IV) non‐small cell lung cancer (NSCLC) between January 2005 and December 2016 at the 14 hospitals of the Chubu Lung Cancer Surgery Study Group. A total of 37 patients were assigned to the salvage surgery group; a lobectomy with mediastinal lymph node dissection was performed. The survival benefit was assessed using the Kaplan–Meier method and the Cox proportional hazard model.

**Results:**

Although postoperative complications were observed in 11 patients (29.7%), surgery‐related death occurred in only one patient (mortality rate: 2.7%) resulting from respiratory failure caused by interstitial pneumonia exacerbation. Postoperative recurrence was observed in 22 patients (61.1%), the incidence of brain metastasis being high (nine patients: 40.9%). The five‐year survival rate from the first day of treatment was 60%. The survival of the postoperative pathological stage (s'‐stage) I group was significantly better (five‐year survival rate: 80.9%) than that of the other groups (*p* < 0.05). S’‐stage was the most significant factor (*p* < 0.01) associated with long‐term survival.

**Conclusions:**

Salvage surgery is a feasible therapeutic modality for advanced lung cancer. Downstaging to s'‐stage I with previous treatment was most important for survival. Complete resection (R0) should be the goal because surgical procedures were tolerated despite intense treatment.

## Graphical abstract

The complication rate after salvage surgery was 29.7%. Salvage surgery for pulmonary lung cancer is safe and may result in long‐term survival, particularly in those with p‐stage I.

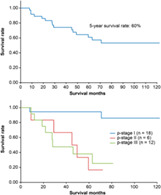

## INTRODUCTION

Recently, much progress has been made in the field of medicine for primary lung cancer. In particular, the advances in efficacy and safety have been astounding. However, a complete cure remains beyond expectations for many patients. Advancements have also been made in supporting therapy for patients undergoing high‐risk surgery, including anesthesia. Based on these circumstances, premeditated induction chemotherapy followed by surgical resection has become an acceptable procedure, being both safe and effective. Similarly, patients with incompletely cured lung cancer after intense medical treatment can be treated with surgical intervention. Generally, salvage surgery is recognized as a surgical treatment performed for patients with residual tumors or recurring tumors even after definitive chemotherapy and/or radiation therapy. Recently, salvage surgery trials have been attempted in many research institutes, but conclusive findings are still very few. Therefore, the benefits of the procedure are not clarified, and precise guidelines have not been established. This study aimed to demonstrate the safety and efficacy of salvage surgery for patients with advanced lung cancer.

## METHODS

A retrospective cohort study on salvage surgery was conducted in 14 participating institutes from the Chubu Lung Cancer Surgery Study Group. This study was approved by the Institutional Review Board of Mie Chuo Medical Center (MCIRB‐201736, MCCOI‐201736) and affiliated institutes. The requirement for informed consent was waived owing to the retrospective nature of this study. A total of 10 033 patients underwent surgical treatment for primary lung cancer between January 2005 and December 2016. Of these surgeries, 37 (0.37%) were recognized to be salvage surgeries. Herein, a salvage surgery was defined as the surgical resection of remnant or recurrent lung tumors after definitive medical treatment (which included chemotherapy and chemoradiotherapy) that intended radical cure for advanced (clinical stage [c‐stage] III and IV) non‐small cell lung cancer (NSCLC). Cases of surgery for second lung cancer, metastatic recurrence, and surgery after scheduled induction therapy (neoadjuvant surgery) were excluded. The initial staging was performed using computed tomography (CT) with or without fluorodeoxyglucose positron emission tomography (FDG‐PET). Pathological confirmation of metastasis was not compulsory because this was a retrospective cohort study based on everyday medical practice.

The median age was 60 years (range, 38–77 years); six patients (16.2%) were female. Histological subtypes included adenocarcinoma, squamous cell carcinoma, and large cell carcinoma in 30, four, and two patients, respectively. One patient had an undefined morphological pattern of NSCLC. The c‐stage was IIIA, IIIB, and IV in 16, nine, and 12 patients, respectively. The medical treatment included chemotherapy and chemoradiotherapy in 23 and 14 patients, respectively. Of the 37 patients, 33 were treated with platinum‐based chemotherapy (platinum doublet or triplet). Other modalities without platinum included pemetrexed single‐agent therapy, vinorelbine single‐agent therapy, and epidermal growth factor receptor‐targeted tyrosine kinase inhibitors (EGFR‐TKI) single‐agent therapy. In 14 patients treated with chemoradiotherapy, the radiation dose was >60 Gy in 10 patients and >40 Gy in three patients. In one patient using EGFR‐TKI, radiation was restricted at 30 Gy.

Indications for salvage surgery have also been evaluated in the clinical setting without the use of tissue specimens. Cancer viability was assessed using CT alone in patients with recurrent tumors. For patients with remnant tumors, assistance with FDG‐PET was necessary, which revealed increased uptake of FDG in the tumor. In this evaluation, cases of N2 or N3 disease (including severe N1 disease that would be resectable only by total pneumonectomy), cases with T3 or T4 disease, and cases with metastasis were rejected before surgery. The case of complete response regarding mediastinal lymph nodes and/or metastatic lesions was included. Cases with severe complications that were supposed to be dangerous for surgical procedures (poor surgical tolerance) were excluded. Therefore, pure resectable patients became candidates for salvage surgery. Among them, many patients refused surgery, of course, for various reasons. Finally, 37 patients underwent surgery. The total number of patients could not be defined because this study was designed for patients who underwent salvage surgery and to evaluate the safety and benefit of the surgical intervention.

The average time between initial therapy and salvage surgery was 23.1 ± 27.1 months (mean ± SD). The intended surgical procedure was lobectomy with mediastinal lymph node dissection according to the guidelines of the Japanese Lung Cancer Society.^1^ This was accomplished in 32 patients. In three patients, total lung resection was necessary. In two patients, resection was minimized to segmentectomy or wedge resection. TNM stage was re‐estimated pathologically after the operation (s'‐stage).

**Statistical analysis**

TNM staging was based on the seventh edition. Overall survival (OS) was calculated in months from the first day of medical treatment or the day of salvage surgery. The significance of the differences between the two groups was evaluated using the χ^2^‐test and Fisher's exact method. Statistical significance was defined as *p* < 0.05. Multivariate Cox proportional hazard model analysis was used to assess factors associated with long‐term survival: patient age, surgical curability, and s'‐stage. Regarding the curability, R0 was defined as “1”, and non‐R0 (R1 and R2) was defined as “0”. Regarding the overall therapeutic process, c‐stage factors on the first day of medical treatment were also introduced. All statistical analyses were performed using the Excel statistical software package (Excel Statistics 2010).

## RESULTS

Complete resection (R0) was achieved in 35 patients. Two patients were defined as R1 resection by postoperative pathological evaluation because of surgical margin problems. The average surgical time was 296.7 ± 108.8 min (mean ± SD), and average blood loss was 498.3 ± 704.6 ml (mean ± SD). The average length of hospital stay was 18.9 ± 12.0 days (mean ± SD). Perioperative complications were observed in 11 patients (29.7%). Prolonged air leakage (two patients, 5.4%) and arrhythmia (three patients, 8.1%) were the most frequently observed complications. Other complications included pulmonary infarction, chylothorax, and respiratory failure in one patient. No patient required any additional surgical procedure. Surgery‐related death was observed in one patient (mortality rate: 2.7%) who died from respiratory failure caused by exacerbation of interstitial pneumonia on the 31st postoperative day.

Among the 36 patients who tolerated surgery, s'‐stage was I in 18 (including a case of Ef3, i.e., no remnant cancer cells), II in six, and III in 12. Postoperatively, 12 patients received chemotherapy, but this number is not suitable for statistical analysis because of few number. With regard to prognosis, 14 patients (38.9%) died of cancer, and eight (22.2%) were alive bearing cancer, while 14 (38.9%) were cancer‐free. In other words, postoperative recurrence was observed in 22 patients (61.1%). Locomediastinal recurrence was observed in nine patients (25%), including malignant pleuritis and lymphangitis (in one patient). Regarding distant metastasis, the incidence of brain metastasis was extremely high (nine patients; 25%), whereas other distant metastases, i.e., lung and bone metastases, were observed in two patients, respectively.

**FIGURE 1:**
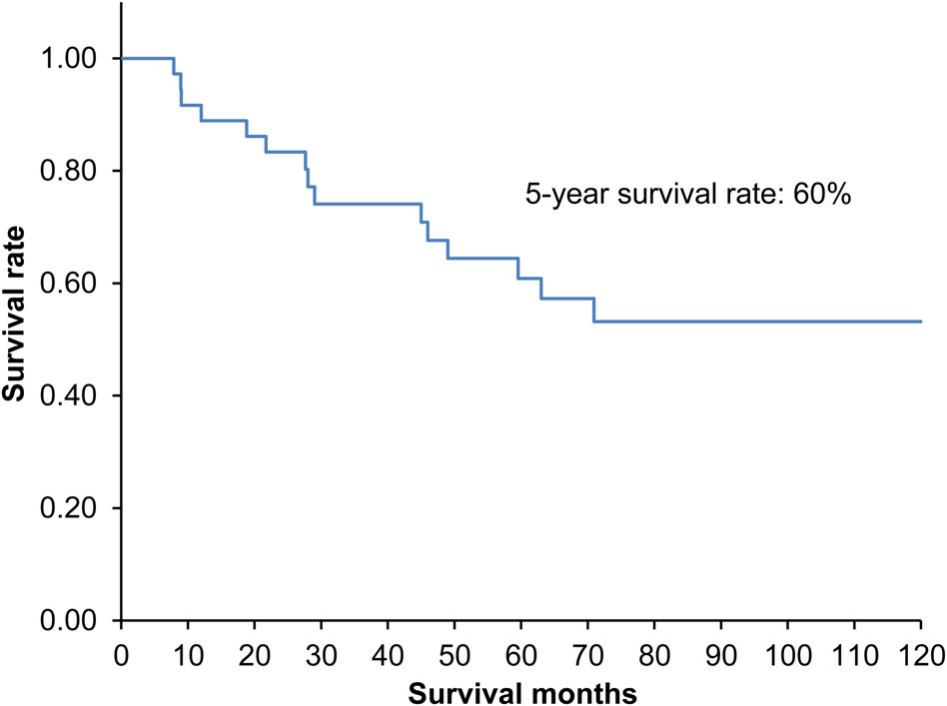
Overall survival after initial treatment in all cases (*n* = 37)

**FIGURE 2:**
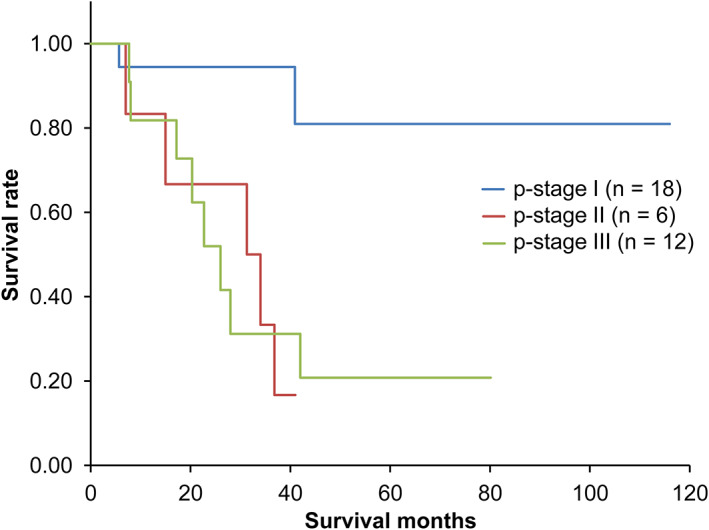
Postoperative overall survival stratified with the postoperative pathological stage (*n* = 36)

**FIGURE 3:**
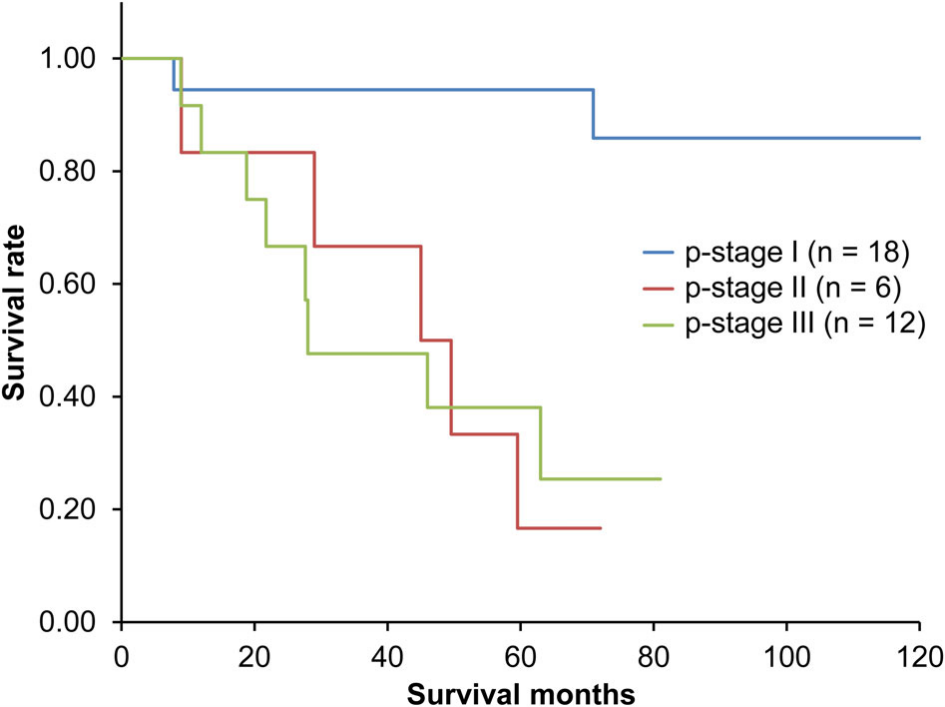
Overall survival from the initial treatment stratified with the postoperative pathological stage (*n* = 36)

The OS curve from the first day of treatment is shown in Figure 1. The five‐year survival rate was 60%. Curves after salvage surgery, which is stratified with the s'‐stage, are shown in Figure 2. The five‐year survival rates were 80.9%, 16.7%, and 20.8% in s'‐stage I, s'‐stage II, and s'‐stage III, respectively. A significant difference was confirmed between the s'‐stage I group and others (*p* < 0.05). OS curves from the first day of treatment are shown in Figure 3. The five‐year survival rates were 94.4%, 16.7%, and 38.2% in s'‐stage I, s'‐stage II, and s'‐stage III, respectively. Survival of the s'‐stage I group patients was significantly better than that of others (*p* < 0.01). Downstaging to s'‐stage I was the most important factor for good survival.

Regarding the factors associated with good postoperative survival, the results of the Cox analysis are shown in Table 1. The s'‐stage was the most significant factor (*p* < 0.01). Regarding the factors associated with good survival of the overall therapeutic process, the results of the Cox analysis are shown in Table 2. The s'‐stage was the most significant factor (*p* < 0.005).

**TABLE 1:** Factors associated with long‐term survival after surgery (Cox analysis)

Covariance	Coefficient	Hazard ratio	*p*‐value
Patient age (years)	−0.0036	0.9964	0.8836
Curability (R0:1; R1, 2:0)	−1.2673	0.2816	0.2752
S’‐stage (postoperatively)	0.8667	2.3790	0.0058*

^*^ *p* < 0.01.

**TABLE 2:** Factors associated with long‐term survival after initial treatment (Cox analysis)

Covariance	Coefficient	Hazard ratio	*p*‐value
Patient age (years)	−0.0088	0.9912	0.7311
Curability (R0:1; R1, 2:0)	−0.5938	0.5897	0.5987
C‐stage (initial stage)	−0.1788	0.8363	0.7812
S’‐stage (postoperatively)	0.9850	2.6777	0.0032*

^*^ *p* < 0.01.

## DISCUSSION

Regarding the safety of salvage surgery, in a study by Sonobe et al.,^2^ no 30‐day postoperative mortality was observed in 29 patients, but combined resection was required for 59% of the patients. However, Romero‐Vielva et al.^3^ reported two cases of a severe complication (bronchopleural fistula) and one case of early postoperative death in a cohort of 27 patients (mortality rate, 3.7%). In the present study, all except one patient tolerated surgery, and the mortality rate was 2.7%. The cause of death was the exacerbation of interstitial pneumonia, which is a common complication, even in routine pulmonary surgery, and is not specific to salvage surgery. However, the use of EGFR‐TKI in preoperative treatment may trigger the occurrence of interstitial pneumonia.^4^ Intense postoperative care is necessary in such cases. Other complications in our study population were mild and could easily be managed. Consequently, salvage surgery was considered safe and feasible.

In this study, the accurate assessment of the efficacy of salvage surgery is impossible because this is a retrospective cohort study conducted by surgeons belonging to the Department of General Thoracic Surgery. The appropriate control group could not be set up in this study. However, almost all patients were incompletely cured, apart from four whose Ef3 was confirmed pathologically after surgery. Accordingly, the expected five‐year survival rate was as low as 10% based on the estimation that 33 patients (four confirmed to be cancer‐free) would not live for more than five years in this study period because the efficacy of second‐ and third‐line chemotherapy had been evaluated based on progression‐free but not on overall survival. A five‐year survival rate of 60% can be considered a benefit of salvage surgery. Sonobe et al.^2^ reported the five‐year relapse‐free survival after initial treatment to be 61%. Uramoto et al.^5^ reported equivalent outcomes of patients with locally advanced NSCLC treated with salvage surgery compared with induction chemotherapy followed by surgical resection. The five‐year OS rates were 65.2% in the induction chemotherapy group and 62.2% in the salvage surgery group. These data are reasonable because neoadjuvant surgery (i.e., induction chemotherapy followed by surgical resection) and salvage surgery both serve the purpose of removing the residual tumor; the former is in an active manner and the latter in a passive one.

The survival curve was significantly better in the s'‐stage I group. Cox analysis also revealed s'‐stage as the most important prognostic factor, implying that downstaging to as low as s'‐stage I during the initial treatment is the most important factor. Further, Sonobe et al.^2^ described that a good response to initial treatment showed a favorable prognosis. However, Renaud et al.^6^ reported that mediastinal downstaging after induction therapy is not a significant prognostic factor in patients with persistent N2 NSCLC. This conclusion was reached because pN1 patients with a high (>1/3) lymph node ratio (number of cancer‐positive lymph nodes over the number of resected lymph nodes) had the same OS as pN2 patients with a low (<1/3) lymph node ratio. However, they also performed a multivariate analysis that showed that mediastinal downstaging (i.e., pN after surgery) is a significant (*p* < 0.0001) prognostic factor, as well as lymph node ratio (*p* = 0.001). Although our study included no data about lymph node ratio, there is no reason to dismiss this finding.

In an ordinary surgical treatment, curative resection (R0) is an important prognostic factor. It is also true in surgeries for N2 NSCLC.^7^ However, curability was not selected as a significant factor in our study. R0 resection is important: no one can survive with remnant cancer in their bodies. It is possible that the small number of non‐R0 patients may have influenced the result. In any event, the power of preoperative medical treatment contributed to a good prognosis, greater than curability. The combination of a powerful medical treatment and minimally invasive surgery may provide a new era of advanced lung cancer therapy. A limitation of this study is the lack of clarity in the surgical indication and the low number of target cases.

In conclusion, salvage surgery is a safe and feasible modality for advanced lung cancer therapy. Postoperative complications were observed in 11 patients (29.7%), and surgery‐related death occurred in only one patient (mortality rate: 2.7%). Moreover, postoperative recurrence was observed in 22 patients (61.1%), and there was a high incidence of brain metastasis (nine patients: 25%). Downstaging to s'‐stage I with previous medical treatment was the most important factor for good survival. Complete resection (R0) should be performed because ordinary surgical procedures are tolerable even after intensive treatment. Salvage surgery will provide patients with advanced lung cancer a chance to achieve long‐term survival.

## CONFLICT OF INTEREST

This research did not receive any specific grant from funding agencies in the public, commercial, or not‐for‐profit sectors. The authors report no proprietary or commercial interest in any product mentioned or concept discussed in this article.
